# Lipopolysaccharide downregulates the expression of ZO-1 protein through the Akt pathway

**DOI:** 10.1186/s12879-022-07752-1

**Published:** 2022-10-05

**Authors:** Peicen Zou, Fan Yang, Yijun Ding, Di Zhang, Ying Liu, Jinjing Zhang, Dan Wu, Yajuan Wang

**Affiliations:** 1 Capital Institute of Pediatrics, Beijing, China; 2 Department of Neonatology, Beijing Children’s Hospital, Capital Medical University, National Center for Children’s Health, Beijing, China; 3 Department of Neonatology, Children’s Hospital, Capital Institute of Pediatrics, Beijing, China

**Keywords:** LPS, Blood-brain barrier, Tight junction protein, PI3K/Akt signal pathway

## Abstract

**Background:**

Neonatal bacterial meningitis is a common neonatal disease with high morbidity, and can cause serious sequelae when left untreated. *Escherichia coli* is the common pathogen, and its endotoxin, lipopolysaccharide (LPS) can damage the endothelial cells, increasing the permeability of the blood-brain barrier (BBB), leading to intracranial inflammation. However, the specific mechanism of bacterial meningitis induced by LPS damaging BBB remains unclear. In this study, the mouse brain microvascular endothelial (bEND.3) cells were used as a research object to investigate whether LPS damage BBB through the PI3K/Akt pathway.

**Methods:**

The bEND.3 cells were stimulated with different concentrations of LPS for 12 h, and the expression of tight junction proteins (ZO-1, claudin-5, occludin) was detected using western blotting. The cells were challenged with the same concentration of LPS (1ug/ml) across different timepoints (0, 2 h, 4 h, 6 h, 12 h, 24 h). Expression of TJ proteins and signal pathway molecules (PI3K, p-PI3K, Akt, p-Akt) were detected. The distribution of ZO-1 in bEND.3 cells were detected by immunofluorescence staining.

**Results:**

A negative correlation is observed between ZO-1 and LPS concentration. Moreover, a reduced expression of ZO-1 was most significant under 1 ug/ml of LPS, and the difference was statistically significant (*P* < 0.05). Additionally, there is a negative correlation between ZO-1 and LPS stimulation time. Meanwhile, the expression of claudin-5 and occludin did not change significantly with the stimulation of LPS concentration and time. The immunofluorescence assay showed that the amount of ZO-1 on the surface of bEND.3 cells stimulated with LPS was significantly lower than that of the control group. After LPS stimulation, p-Akt protein increased at 2 h and peaked at 4 h. The titer of p-PI3K did not change significantly with time.

**Conclusion:**

LPS can downregulate the expression of ZO-1; however, its effect on claudin-5 and occludin is minimal. Akt signal pathway may be involved in the regulation of ZO-1 expression induced by LPS in bEND.3 cells.

**Supplementary Information:**

The online version contains supplementary material available at 10.1186/s12879-022-07752-1.

## Introduction

Neonatal bacterial meningitis is the most common and serious life-threatening infectious disease of the central nervous system in pediatrics, and has a high incidence and mortality rate [1, 2]. Many pathogenic bacteria can cause neonatal bacterial meningitis. Concurrently, the use of antibiotics, vaccines, as well as changes in the natural environment can lead to changes in the dominant pathogenic bacteria. These results to emergence of many drug-resistant bacteria, especially multi-drug-resistant strains, bringing difficulties in the clinical treatment of neonatal bacterial meningitis. The coagulase-negative Staphylococcus and *Staphylococcus aureus* were the dominant pathogens of neonatal suppurative meningitis in the 1970 and 1980 s [3, 4], which were replaced by *Group B Streptococcus* (*GBS*) and *Escherichia coli* (*E. coli*) in the 1990s. With monitoring and management during pregnancy, the incidence of GBS meningitis decreased significantly in developed countries, and studies have shown that *E. coli* is the dominant pathogen of neonatal bacterial meningitis [1, 3, 5] in recent years. Paying attention to neonatal *E. coli* infection has become the focus of reducing the occurrence and development of neonatal infectious diseases and neonatal mortality.

The blood-brain barrier (BBB) is composed of the brain microvascular endothelial cells and their tight junctions (TJ), which mainly served as a physical barrier [6]. TJ consists of a variety of protein complexes, including transmembrane proteins, cytoplasmic adhesion proteins, junction adhesion molecules, and cytoskeleton proteins. Occludin is an integral membrane protein within the TJ of endothelial cells, and has four transmembrane domains and two extracellular loops [7]. The claudin protein family has a total of 27 family members, all of which share the same protein pattern: four membrane-spanning domains, two extracellular loops, and two cytoplasmic-terminal intact membrane proteins (short-chain N-terminal and longer-chain C-terminal) [8]. Zonula occludens protein 1 (ZO-1) is a member of the membrane-associated guanosine monophosphate kinase family and is an important scaffold protein that connects TJ proteins and actin cytoskeletons [9]. Among these, occludin and claudins protein families are transmembrane proteins. These transmembrane proteins are connected to the cytoskeleton through the cytoplasmic attachment protein ZO, forming a complete junction structure [10]. TJ is the material basis for maintaining the basic structural and functional integrity of BBB [11]. When the body is infected by bacteria, it can destroy the TJ protein, and decrease its expression, leading to the destruction of the BBB function, and production of an inflammatory response in the brain.

The regulation of TJ protein is closely related to signaling pathways. Guo et al. [12] reported that lipopolysaccharide (LPS) regulates the permeability of TJ by activating the TLR4 signaling pathway in the FAK and MyD88 signaling pathways. The TLR4/FAK/MYD88 signaling axis is formed by activating IRAK-4, which ultimately leads to the downregulation of TJ proteins. Sharif et al. [13] proposed that activation of RhoA/ROCK/MLC signaling pathway could destroy the integrity of TJ. However, the signaling pathways affecting claudins are complex. Presently, some studies reported that NF-κB, p38/MAPK, and Akt signaling pathways may mediate the expression of TJ proteins [14, 15]. Each signal pathway interleaves and influences the other. Among them, the PI3K/Akt signaling pathway plays an important role in the regulation of BBB. One study reported that knocking-out PI3K gene could reduce the damage of BBB, and decrease the expression of claudin-5 in mice [16]. Akt is the key molecule of the PI3K signal transduction pathway and is related to endothelial cell function, angiogenesis, and vascular physiology [17]. Butyrate increases the expression of TJ protein in intestinal epithelial cells by activating Akt/mTOR-mediated protein synthesis [18]. Meanwhile, COM crystals lead to TJ breakage of distal renal tubular epithelial cells by activating ROS/Akt/p38MAPK pathway [19]. When *E. coli* forms sepsis in vivo, the bacteria can destroy the intercellular connection structure, downregulate the concentration of TJ protein in endothelial cells, cross BBB, stimulate the release of inflammatory factors in the body, and eventually lead to intracranial infection [20, 21]. Therefore, this study speculates that *E. coli* regulates the expression and phosphorylation of TJ protein through the PI3K/Akt signaling pathway, which may provide a new target for the treatment of bacterial meningitis.

LPS as the endotoxin of *E. coli* is the key factor leading to *E. coli* meningitis. In this study, we used bEND.3 (brain microvascular endothelial) cells as the research object to understand the effect of LPS on the expression of TJ protein, and explore its correlation with PI3K/Akt signal pathway, as well as further clarify the pathological mechanism of neonatal bacterial meningitis.

## Materials and methods

### Cell culture

The bEND.3 cells were grown in a complete medium in 37 ℃ incubator under 5% CO_2_: DMEM high glucose medium (89%), newborn bovine serum (10%), penicillin-streptomycin antibiotic solution (1%). The culture medium was replaced approximately 2–3 days according to cell growth.

### LPS challenge

The bEND.3 cells were spread on a six-well plate and adhered to the wall. When the cells covered the bottom of the board to 90%, the fresh medium was added, and the cells were randomly divided into two groups. Subsequently, the cells were started to challenge with LPS. In contrast, different concentrations of LPS (10 ng/ml, 1 ug/ml) were added to the six-well plate with the same amount of culture medium challenged for 12 h. Meanwhile, the bEND.3 cells were challenged with the same concentration of LPS (1ug/ml) across different times (0, 2 h, 4 h, 6 h, 12 h, 24 h). The culture medium was then removed and the basal cells were preserved. Western blotting was performed to detect the expression of ZO-1, Claudin-5, Occludin, and β-actin in both groups.

### Western blot

The cell was scraped in cell lysate and centrifuged at 12,000 rpm for 10 min at 4℃. The supernatants were collected and the protein concentration was measured using the BCA method. The protein was denatured in a metal bath at 100 °C for 10 min. The loading volume of each empty sample was 15 µg, and the protein was separated by 12% SDS-PAGE and was subsequently transferred to the PVDF membrane. The membrane was blocked in FBS solution for 1 h and the antibodies ZO-1, claudin-5, occludin, β-actin (Invitrogen, 1:2000); PI3K, p-PI3K, Akt, p-Akt (CST, 1:2000) were incubated at 4 °C overnight. The secondary antibody was incubated the next day at room temperature for 1 h. The target band was placed directly on the Biorad developing instrument, and the image of the developed band was saved. The gray value of the band was determined by Image J, and the content of the target protein was reflected by the ratio of the gray value of the target protein to the gray value of the internal reference protein.

### Immunofluorescence

The cells were stimulated with 1 ug/ml LPS for 6 h, fixed with 4% PFA solution for 10 min at room temperature, washed with PBS three times, and were blocked with 3% BSA for 30 min at room temperature. The antibodies ZO-1, claudin-5, occludin (1:400) were incubated overnight. After washing three times with PBS, the cells were incubated with the fluorescently labeled secondary antibody for 1 h at 37 °C. The nucleus was stained with DAPI and incubated at room temperature for 5 min, and the slides were mounted with glycerol and photographed using a fluorescence microscope (Olympus).

### Statistical analysis

This experiment was independent, and all experiments were repeated three times. The software SPSS version 19.0 (IBM Corp., Armonk, NY, USA) was used for the statistical analysis. Measurement data were described as means ± SE, and were analyzed using a one-way ANOVA test. A *P* < 0.05 difference was considered as statistically significant.

## Results

### Changes in TJ protein expression when challenged with different concentrations of LPS

**Fig. 1:**
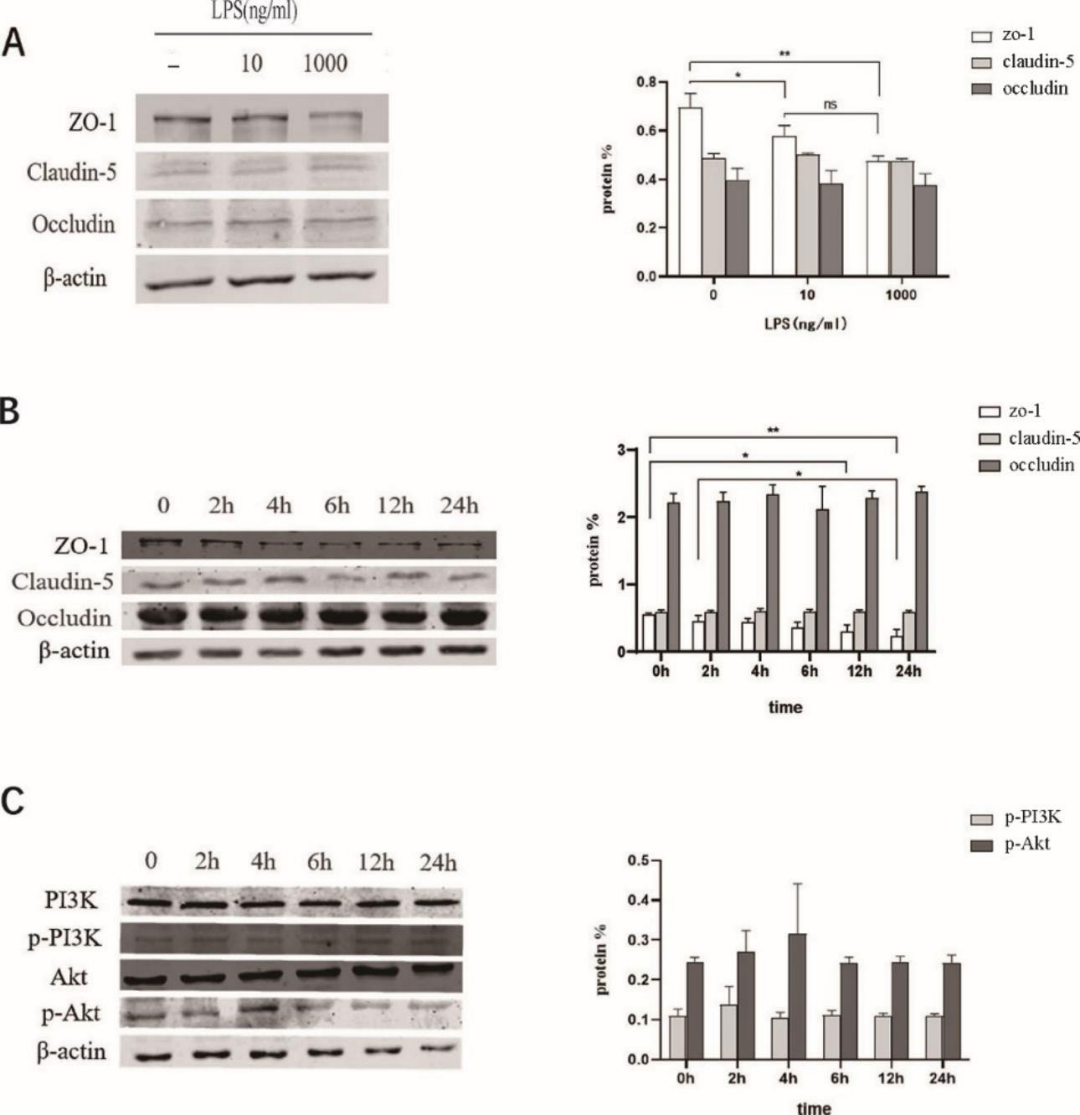
Stimulation of bEND.3 cells with LPS. **(A)** Changes of the expression of TJ protein stimulated with different concentrations of LPS at the same time (12 h). The expression of ZO-1 was downregulated in a concentration-dependent manner, which is more apparent under 1 ug/ml of LPS concentration. The expression of claudin-5 and occludin did not change significantly with the stimulation of LPS concentration. **(B)** Same concentration of LPS (1ug/ml) stimulated at different timepoints (0, 2 h, 4 h, 6 h, 12 h, 24 h) closely changes the expression patterns of TJ protein. The expression of ZO-1 was time-dependent and decreased most significantly after 24 h. The expression of claudin-5 and occludin did not change significantly with time. **(C)** Changes of PI3K/Akt signaling pathway proteins in LPS-stimulated cells. The p-Akt protein increased after 2 h and peaked after 4 h. ns, not statistically significant; *, *P* < 0.05; **, *P* < 0.01.

The results showed that the expression of ZO-1 was downregulated in a concentration-dependent manner. The higher the concentration of LPS, the lower the gray value and ZO-1 expression. The expression of ZO-1 was most significantly downregulated under 1 ug/ml concentration of LPS, and the difference was statistically significant (*P* < 0.05). The expression of claudin-5 and occludin did not significantly change with the stimulation of different LPS concentrations (Fig. 1 A).

### Changes in the expression of TJ protein when challenged with the same concentration of LPS across different timepoints

We detected the expression of TJ proteins (ZO-1, claudin-5, occludin protein) after challenging the cells with the same concentration of LPS (1 ug/ml) across different timepoints (0, 2 h, 4 h, 6 h, 12 h, 24 h). We found that the expression of ZO-1 was downregulated in a time-dependent manner. The longer the LPS stimulation time, the lower the gray value and the expression of ZO-1. The expression of ZO-1 decreased most significantly after 24 h, and the difference was statistically significant (*P* < 0.05). However, the expression of claudin-5 and occludin did not change significantly with time (*P* > 0.05) (Fig. 1B).

### Changes in PI3K/Akt signaling pathway protein expression in LPS-stimulated cells

The protein expression levels of PI3K, p-PI3K, Akt, and p-Akt in the PI3K/Akt signaling pathway were detected. Although p-PI3K did not change significantly over time, p-PI3K and p-Akt initially increased and then decreased gradually, with p-PI3K peaking at 2 h and p-Akt peaking at 4 h (Fig. 1 C). Taken together, these data suggested that Akt may affect the expression of TJ protein.

### LPS decreased the amount of ZO-1 protein on the membrane of bEND.3

**Fig. 2:**
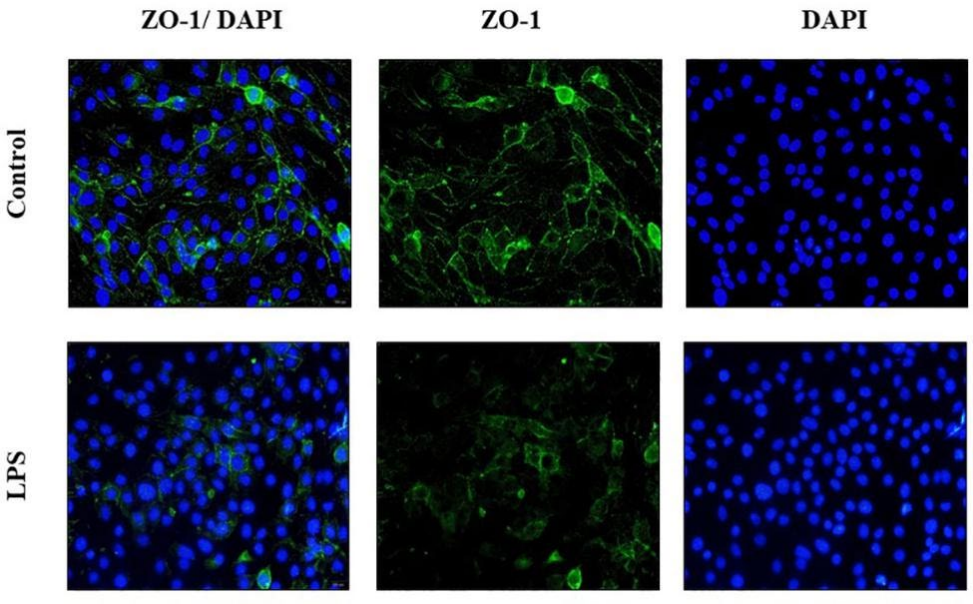
LPS reduced the expression of ZO-1 in bEND.3 cells

Since the expression of ZO-1 decreased significantly with the increase of LPS dose and stimulation time after the LPS challenge, we used immunofluorescence to detect the distribution of ZO-1 in bEND.3 cells stimulated with LPS (1ug/ml) for 6 h. Compared with the control group, the amount of ZO-1 on the surface of bEND.3 cells decreased significantly (Fig. 2), indicating that LPS can reduce the expression of tight junction protein.

## Discussion

Neonatal bacterial meningitis is a serious life-threatening infectious disease of the central nervous system in pediatrics. The incidence of neonatal bacterial meningitis accounts for approximately 0.2– 6.1‰ of live births [3, 5]. The clinical manifestations of neonatal bacterial meningitis are atypical, and thus early diagnosis can be challenging and can affect timely treatment. The incidence of *E. coli* meningitis especially among late newborns, increased annually in recent years. Due to atypical clinical symptoms, life-threatening conditions and highly-drug-resistant nature of neonatal *E. coli* bacterial meningitis [22], understanding the molecular mechanism of the disease and finding a therapeutic direction are necessary.

The BBB is formed by brain microvascular endothelial cells, and the TJ seals the intercellular space and form a physical barrier. TJ is a complex formed by a variety of TJ proteins, which are essential for the integrity and function of the BBB. Damage to the BBB allows pathogenic bacteria to penetrate the brain, which can lead to bacterial meningitis. Different pathogenic bacteria can damage the BBB through various mechanisms; however, the molecular mechanism of the damage of *E. coli* to the BBB by destroying TJ has not been fully elucidated [23].

In this study, we detected the expression of TJ proteins including, ZO-1, occludin, and claudin-5. These proteins can interact with cytoskeleton scaffold proteins to form atresia bands in cells and strengthen the connection between cells [24]. Studies have shown that a decrease in TJ integrity can lead to the destruction of the BBB. Loss in barrier integrity allows antigens (microorganisms, toxins) in the blood vessels to enter the brain through the intercellular space, consequently destroying the homeostasis of the intracranial environment, leading to inflammatory response [25]. Some pathogens can cause vascular endothelial cell permeability defects by changing the integrity of TJ [26]. LPS is an endotoxin of *E. coli*, which can destroy the barrier function of microvascular endothelial cells [27]. To clarify the molecular mechanism of *E. coli* passing through BBB, this study used LPS as a stimulator to understand the expression pattern of TJ proteins in bEND.3 cells, and explore the effect of the PI3K/Akt signaling pathway on cell-cell connections.

bEND.3 cells are vascular endothelial cells commonly constituting the BBB in mice, and is often used in studying BBB in vitro. Lanhui et al. used LPS to stimulate human brain microvascular endothelial cells and found a reduced expression of ZO-1 and occludin, suggesting that TJ proteins play a key role in mediating BBB permeability [28]. In this experiment, we stimulated bEND.3 cells with 10 ng/ml and 1 ug/ml concentrations of LPS for 12 h, and found that the amount of TJ proteins on the cell membrane decreased, especially the expression of ZO-1. The higher the concentration of LPS, the less the amount of ZO-1 on the cell membrane. When 1 ug/ml of LPS was used to stimulate bEND.3 cells, the expression of ZO-1 decreased significantly. However, the expression of occludin and claudin-5 protein did not decrease significantly, which can be attributed to the different research cells selected in this experiment. Dong et al. found that the protein levels of claudin, occludin, and ZO-1 are negatively correlated with the mRNA levels of IL-6, IL-1 β, and TNF- α in mice infected with bacteria [29]. Zhao et al. [30] stimulated human umbilical cord microvascular endothelial cells with different concentrations of LPS in vitro, and found that the content of intracellular reactive oxygen species (ROS) increased significantly. Moreover, the TJ between cells was destroyed, and the amount of TJ protein on the cell membrane decreased. In this experiment, the concentration of ZO-1 protein decreased with the increase of LPS. After LPS stimulation, cells produced many inflammatory factors and ROS; the higher the concentration of LPS, the more inflammatory factors and ROS were produced. Thus, the production of TJ protein was inhibited. We performed cellular immunofluorescence on LPS stimulated and control cells at the cellular level. Compared with the control group, the immunofluorescence of the cell membrane in the LPS stimulation group decreased significantly, indicating that the expression of ZO-1 on the cell membrane also decreased. The results were consistent with the protein level.

LPS-induced neuroinflammation was not only dose- but also time-related [31]. Biesmans et al. [32] have found that neuroinflammation changed over time and certain symptoms in the brains of mice subsided after 24 h of LPS intervention. In this study, decreased TJ protein was most significant when the cells were challenged with 1ug/ml LPS. To further explore the effect of LPS on the TJ of bEND.3 cells, we stimulated the cells with 1ug/ml LPS across different timepoints, and observed changes in the expression pattern of ZO-1, occludin, and claudin-5, which decreased through time. The expression of ZO-1 was reduced especially when stimulated for 24 h, and the difference was statistically significant (*P* < 0.05). Meanwhile, no significant reduction in the expression of occludin and claudin-5 protein was observed (*P* > 0.05). Fang et al. [33] stimulated the endothelial cells in vitro with 10 ug/ml LPS and found that LPS can downregulate the expression of ZO-1, claudin-5, and occludin. Moreover, the time with the lowest expression level was consistent with the time with the lowest TEER value.

In this experiment, only the ZO-1 protein in TJ decreased significantly, while occludin and claudin-5 protein did not, which can be due to the cell state and LPS concentration in this study. Most ZO-1 are located at the TJ of cells, and the stimulation of LPS can reduce the expression of TJ protein. Studies have shown that vitamin A can reverse the effect of LPS; however, the specific mechanism remains unclear [34].

PI3K is a family of lipid kinases. Under physiological conditions, PI3K is typically activated by extracellular signals, including growth factors, cytokines, and hormones. The activation and stability of Akt are carefully regulated by phosphorylation of a variety of signal molecules [35]. In this experiment, after stimulating bEND.3 cells with 1ug/ml LPS, we found that the expression of p-PI3K did not change significantly; however, p-Akt began to increase after 2 h and peaked at 4 h. Zheng et al. [36] challenged the human lung microvascular endothelial cells with LPS, and found that p-Akt initially increased and then decreased with the stimulation time, which is consistent with our experiments. Moreover, we speculate that the downregulation of the expression of ZO-1 induced by LPS in bEND.3 cells may be related to the phosphorylation of Akt in the signal pathway. Since the expression of ZO-1 protein began to decrease significantly after 2 h of stimulation and reached the peak 12 h after stimulation, it’s expression decreased significantly earlier than that before the translation. However, there was no significant change in p-PI3K after LPS stimulation. It was considered that PI3K signal molecules were not involved in the regulation of TJ protein. Currently the mechanism of how activated Akt regulates TJ protein has not been elucidated. Studies have shown that LPS can downregulate the phosphorylation of Akt and 4e-bp1 in endothelial cells, subsequently damaging the cell barrier. It is suggested that LPS may reduce the density of TJ protein by inhibiting Akt/mTOR-mediated protein synthesis [18]. Yu et al. [19] reported that TJ fracture can be induced by activating the ROS/AKT/p38MAPK pathway. They proposed that ASK1 and p38 MAPK were triggered by generating a large amount of ROS, which activates Akt, leading to a significant decrease in the expression of ZO-1. Inhibition of production of ROS using N-acetyl-L-cysteine can attenuate the activation of Akt, Ask1, p38-MApk, and the downregulation of ZO-1. In this experiment, the downregulation of TJ protein is considered to be caused by LPS activation of Akt, which in turn activates the downstream signaling molecules.

In conclusion, LPS can downregulate the expression of ZO-1 protein in bEND.3 cells in vitro, through the Akt signal pathway.

## Electronic supplementary material

Below is the link to the electronic supplementary material.

Supplementary Material 1

## Data Availability

All data generated and analyzed during this study are included in this published article and its additional information.

## Abbreviations

LPS: Lipopolysaccharide.
BBB: blood-brain barrier.
bEND.3: brain microvascular endothelial cells.
TJ: tight junction.
GBS: Group B Streptococcus.
*E. coli*: *Escherichia coli*.
PI3K: Phosphoinositide 3-kinase.
Akt: protein kinase B.
p-Akt: Phospho-protein kinase B.
ZO-1: zonula occludens protein 1.
MAPK: mitogen-activated protein kinase.
