# Supplementary material for: Lipopolysaccharide downregulates the expression of ZO-1 protein through the Akt pathway
Source: BMC Infect Dis. 2022 Oct 5;22:774. doi: 10.1186/s12879-022-07752-1 (PMC9533599; doi:10.1186/s12879-022-07752-1)
Supplement: Supplementary file 1 — Supplementary Material 1 [file 12879_2022_7752_MOESM1_ESM.pdf]

Lipopolysaccharide downregulates the expression of ZO-1 protein through the Akt pathway

Peicen Zou[1], Fan Yang[2], Yijun Ding[2], Di Zhang[3], Ying Liu[2], Jinjing Zhang[2], Dan Wu[2], Yajuan Wang[3,*]

[1]Capital Institute of Pediatrics, Beijing, China

[2]Department of Neonatology, Beijing Children's Hospital, Capital Medical University, National Center for Children’s Health, Beijing, China

[3]Department of Neonatology, Children's Hospital, Capital Institute of Pediatrics, Beijing, China

[*]Correspondence:

Yajuan Wang

Address: Department of Neonatology, Children's Hospital, Capital Institute of Pediatrics,2#Yabao Road, Chaoyang District 100020, Beijing, China

E-mail: cxswyj@vip.sina.com.

Tel: (+86) 13501341241

Figure 1 A

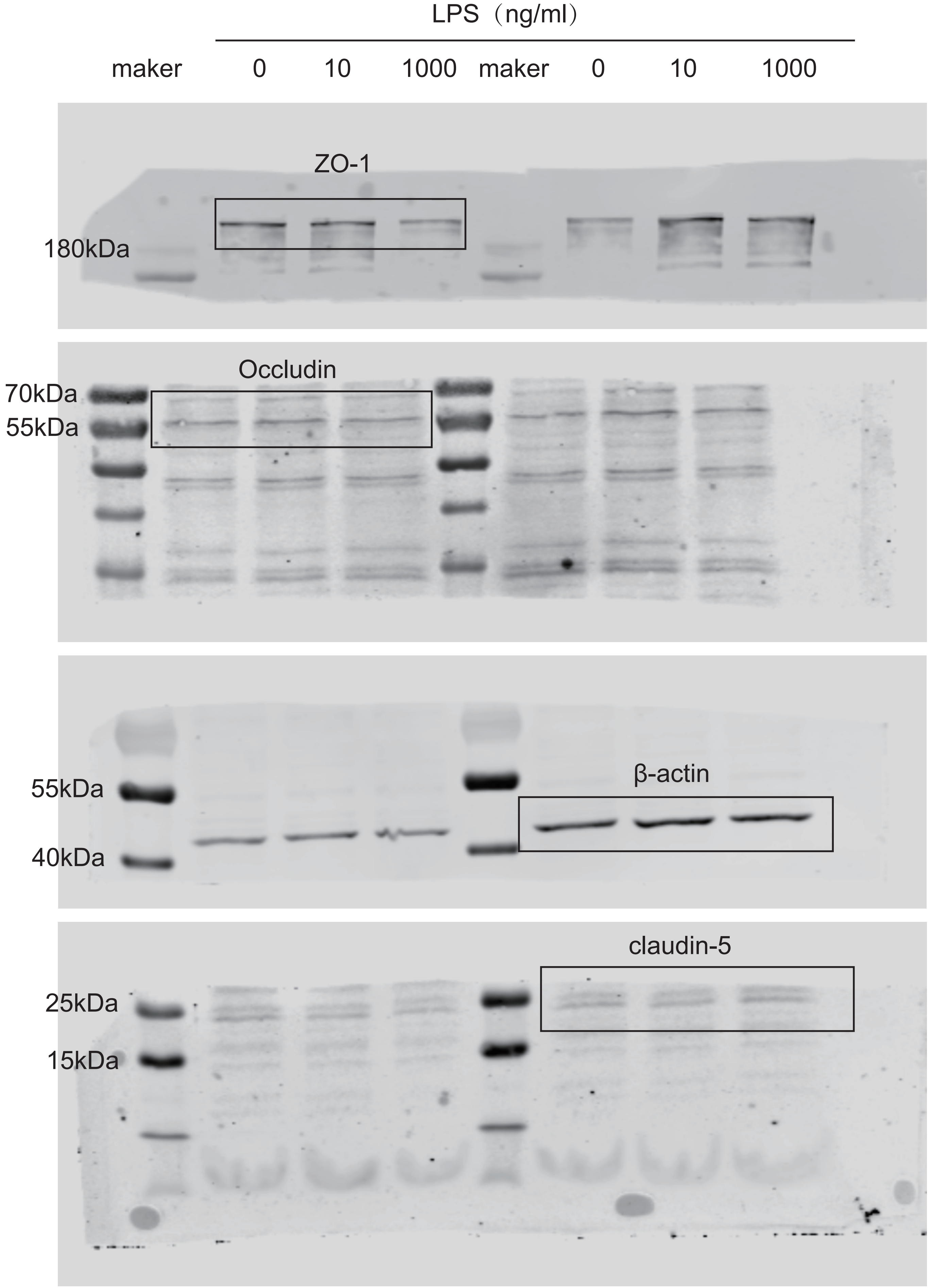
LPS（ng/ml）
maker
0
10
1000
maker
0
10
1000
ZO-1
180kDa
Occludin
70kDa
55kDa
β-actin
55kDa
40kDa
claudin-5
25kDa
15kDa

Figure 1 B

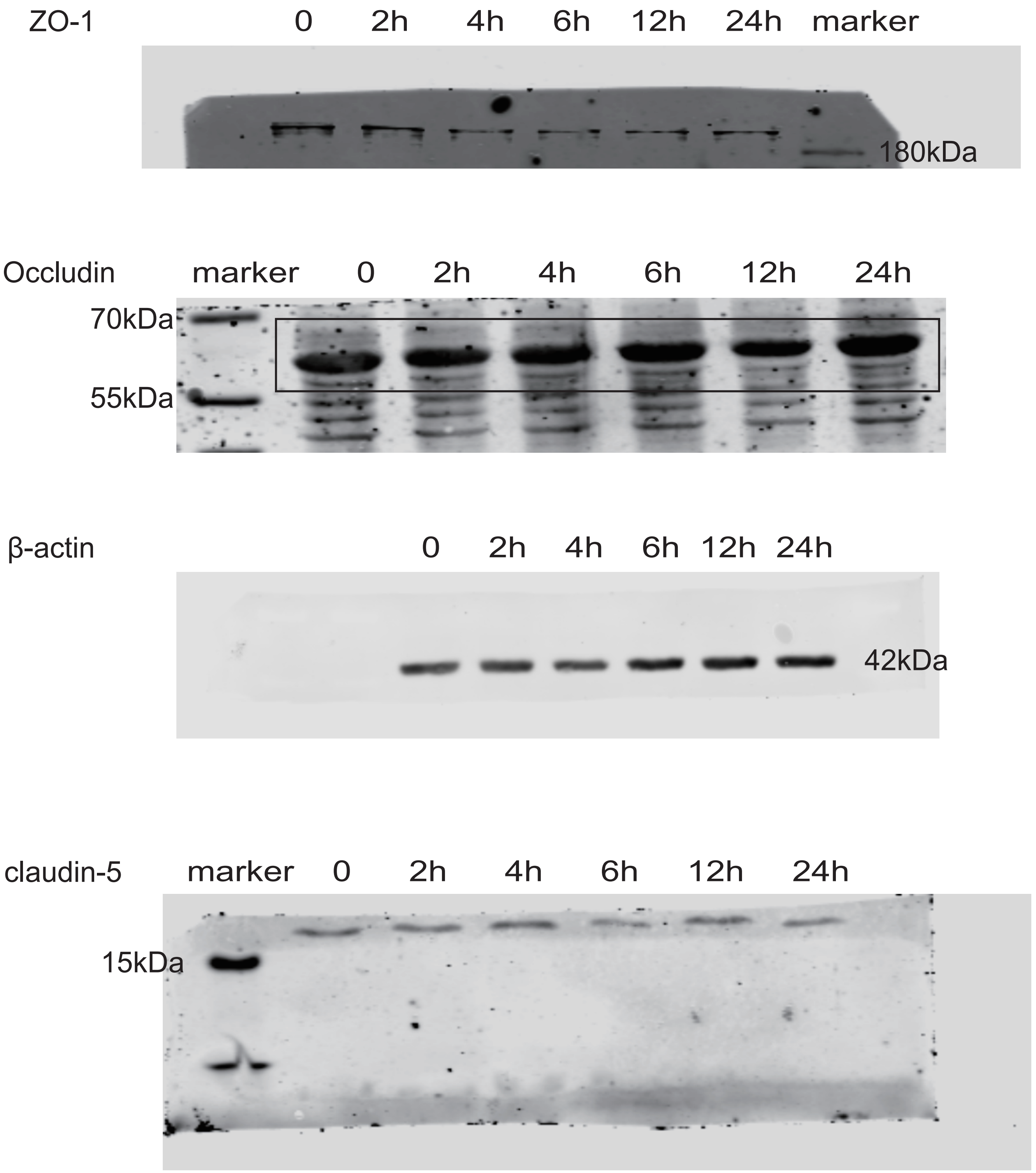

ZO-1
0
2h
4h
6h
12h
24h
marker
180kDa
Occludin
marker
0
2h
4h
6h
12h
24h
70kDa
55kDa
β-actin
0
2h
4h
6h
12h
24h
42kDa
claudin-5
marker
0
2h
4h
6h
12h
24h
15kDa

Figure 1 C

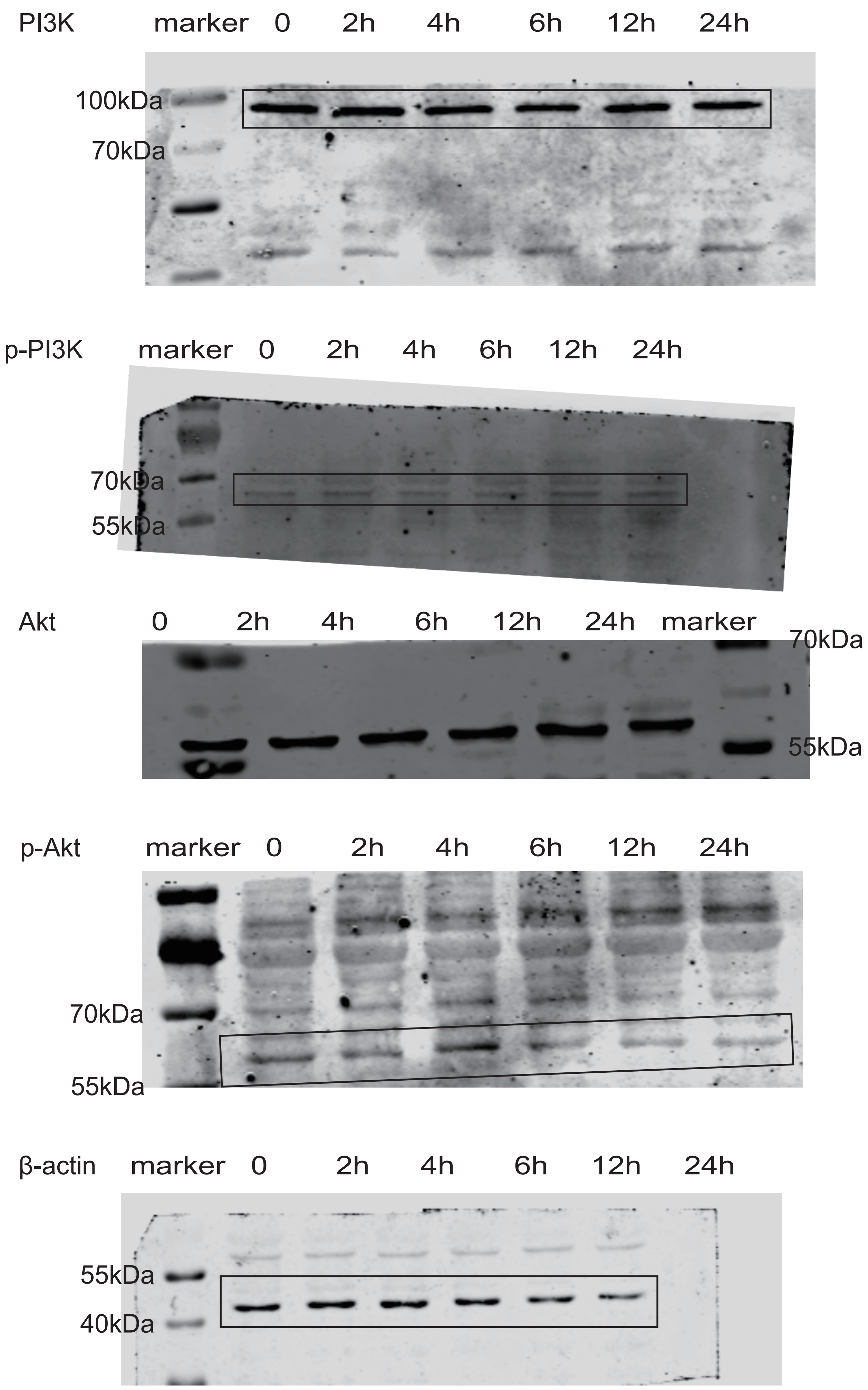
PI3K
marker
0
2h
4h
6h
12h
24h
100kDa
70kDa
p-PI3K
marker
0
2h
4h
6h
12h
24h
70kDa
55kDa
Akt
0
2h
4h
6h
12h
24h
marker
70kDa
55kDa
p-Akt
marker
0
2h
4h
6h
12h
24h
70kDa
55kDa
β-actin
marker
0
2h
4h
6h
12h
24h
55kDa
40kDa

Figure 1. Stimulation of bEND.3 cells with LPS. **(A)** Changes of the expression of TJ protein stimulated with different concentrations of LPS at the same time (12h). The expression of ZO-1 was downregulated in a concentration-dependent manner, which is more apparent under 1 ug/ml of LPS concentration. The expression of claudin-5 and occludin did not change significantly with the stimulation of LPS concentration. **(B)** Same concentration of LPS (1ug/ml) stimulated at different timepoints (0, 2h, 4h, 6h, 12h, 24h) closely changes the expression patterns of TJ protein. The expression of ZO-1 was time-dependent and decreased most significantly after 24 hrs. The expression of claudin-5 and occludin did not change significantly with time. **(C)** Changes of PI3K/Akt signaling pathway proteins in LPS-stimulated cells. The p-Akt protein increased after 2 hrs and peaked after 4h.

ns, not statistically significant; *, $P < 0.05$; **, $P < 0.01$.
